# Quality of life for chronic psychiatric illnesses and home care

**DOI:** 10.12669/pjms.322.8794

**Published:** 2016

**Authors:** Nesibe Gunay Molu, Birgul Ozkan, Sema Icel

**Affiliations:** 1 Nesibe Gunay Molu, Yildirim Beyazit University, Faculty of Health Sciences, Psychiatric Nursing, Yildirim Beyazit University, Ankara, Turkey; 2 Birgul Ozkan, Yildirim Beyazit University, Faculty of Health Sciences, Psychiatric Nursing, Yildirim Beyazit University, Ankara, Turkey; 3 Sema Icel, Yildirim Beyazit University, Faculty of Health Sciences, Psychiatric Nursing, Yildirim Beyazit University, Ankara, Turkey

**Keywords:** Home care, Psychiatric patients, Quality of life

## Abstract

Nowadays, mental illnesses are gradually increasing and so does chronic psychiatric patients. As a result of this increase, chronic psychiatric disorders lead the burden of patients and their families. To reduce the burden of mental illnesses on individuals and their families, treatment and care are given including psychosocial, physiological and medical support and social services. To begin with, home care enables both the patient and his or her family to stay at their own houses and not to be bothered with residents or long-term, institutional-based nursing homes. In addition, the home care providers deliver services to the patient’s at their own house. The other advantages of taking care at home is that it eases financial issues in terms of reducing the cost, reduces the patient’s symptoms and improve the individual’s quality of life (QoL). In addition to these, home care also minimizes the burden on outpatient services and provides help for the patient and the family in order to solve their problems and give support. Home care services help patients to get their freedom back and enhance the quality of their lives. Thus, it is necessary to procure and implement these services and supply both the patient and his or her family a high-quality life.

**Sources of data/ study selection:**

Literature review was done by using the keywords “home care, patient with chronic mental illness, quality of life, home care nursing” from the sources including PsychINFO, PsychARTICLES, MEDLINE, PubMED, EBSCOHOST and The COCHRANE LIBRARY in the time period of 2005- 2015.

## INTRODUCTION

The aim of this study was to examine the impact of patients having psychiatric disease and the care provided to the caregivers in home on the life qualities of the patients and the caregivers.

Nowadays, mental illnesses are gradually increasing. People with mental disorders experience disproportionately higher rates of functional loss and mortality. Mental illnesses in disability is second with 19% in Turkey.^1^ In chronic mental disorders there are a lot of functional losses, which also lead to social and economic losses. A patient with chronic mental illness is unable to fulfill his or her roles and responsibilities, he or she is in need of care and becomes a burden for his or her family.^1^-^4^

Cognitive and functional inabilities in a patient causes behavioral problems and the level of burden of the caregiver is affected by many factors. To reduce the burden of mental illnesses on individuals and their families, certain treatments and care are provided, including psychosocial, physiological and medical support as well as social services. The home care services must be in contact with the patients as well as their families to provide them with problem solving skills and increase their level of tolerance.^3^, ^5^-^8^ While Gellis et al. emphasizes that problem solving therapies improve the quality of life at home care, Belle et al. identified that caregivers’ quality of life can be improved through multiple components.^9^, ^10^ The multiple components which are listed as provision of information, didactic instruction, role-playing, problem-solving, skills’ training, stress management techniques, and tele-support groups aim to reduce risk in the study’s five target areas by providing caregivers with education, skills to manage troublesome care recipient behaviors, social support, cognitive strategies for reframing negative emotional responses, and strategies for enhancing healthy behaviors and managing stress. 9, 10 Muijenet al. stated that home care reduced the duration of the treatment 80% compared to hospital care.^11^

Home care is applicable to individuals at all ages with acute/long-term physical illnesses or psychiatric illnesses. In the case of changes in patients’ mental status, the necessary physical, social and emotional support, preventive, curative, supportive, rehabilitative support, as well as health care and palliative care are provided.^12^-^16^ Home care services provide sustainability of health services and its aim is to develop, sustain, protect and rehabilitate the patient. Home care also aims to enables people to remain at home rather than use residential, long-term, or institution-based nursing homes. Home care providers deliver services in the client’s own home.^17^ Care at home is safer and more effective for patients appropriate for it. It decreases the costs and symptoms and increases patients’ life quality. Home care also decreases the usage of the beds, thereby the period of staying at hospital. Daily tasks including all the homework are procured by life assistance services. After patients’ discharge, home care is often required.^17^, ^18^

### Home care services

The home care services may include some combination of professional health care services and life assistance services. Home care services could include medical or psychosocial assessment, wound care, follow up and treatment of chronic diseases, breathing exercises, physical therapy, medication teaching, pain management, disease education and management, physical therapy, or occupational therapy.^15^, ^16^, ^19^ Life assistance services include help with daily tasks such as meal preparation, medication reminders, errands, shopping, transportation, and companionship. Home care is often an integral component of the post-hospitalization recovery process, especially during the initial weeks after discharge when the patient still requires some level of regular physical assistance.^16^

Home health care services for psychiatric patients should be launched one or two months after being discharged from the hospital and the visits should be once or three times in a week. During houses visits, sincerity of the team is important as a requirement of basic professional limitations.^20^ Patients and their families who need home health care are provided with home care services, visitor nurses, hospitals and nursing services.^20^ In most countries the staff who provides home care has a standard training. However; registered nurses as well as occupational therapists have bachelor’s degrees in most countries. Particularly, it is emphasized that community psychiatric nurses should take at least one year of master’s degree in the field of psychiatry to support patients at home.^5^ Nurses recruited in home care services must have such skills as mental health assessment, psychological education, cognitive behavioral therapy, symptom management, the education of family/caregiver, care management/ coordination.

Nurses who have these skills will provide more organized and efficient care giving programs. Negative regulations and programs of home care services and nurses also affect the patients. In care giving programs, the functional deficiencies and the requirement of the patients levels should be identified. Borowiak and Kostka emphasized that patients living in urban and rural areas, require a specific nursing although they bear some differences according to the regions where they reside, and those people’s nursing care must be determined according to their functional impairment. Appropriate nursing care of patients will be enabled by determination of their needs. These requirements can be determined by different evaluation methods.^21^

### Patients Assessment and Screening Programmes

Functional/neurological/emotional/behavioral assessment of patients is essential with adequate screening programs to the home care services. However, the healthcare services lacks adequate screening programs to identify patients with mental health issues. Home care services’ negative regulations, inadequate evaluation methods and programs also affect negatively the patients.^22^ Therefore home care services are important for quality of life of chronic psychiatric patients and theirs family members.

Home care service that is given to patients according to their determined needs with adequate evaluation methods are based on the development of patients’quality of life by providing them with the opportunity to gain their independence. The implementation of such services and care will improve patients’ quality of life and that of the family members. The evaluation and determination of the factors affecting the quality of life, and the development of health care individuals with chronic mental illness and their relativesplay an importantrole in the formulation of new acceptable strategies.^20^, ^23^-^25^

### Assessment of Quality of Life

According to the World Health Organization (WHO), the quality of life includes distinct domains and indicators such as physical health, psychological health, level of independence, social relations, environmental factors and personal beliefs. The quality of life can be assessed by different means.^26^, ^27^ Belle et al. emphasizes that the quality of life and depression vary among ethnic groups. In addition, Ishak et al. emphasized that when depression is added to psychiatric illness or other medical illnesses, the quality of life reduces, while Sylyia et al. emphasized that low income, severity of depression, the disease burden and other psychosocial stressors cause a reduction in individuals’ functions and deterioration of the quality of life. As seen in the studies, quality of life is influenced by many factors and should therefore be evaluated properly especially in high risk groups.^10^, ^25^, ^28^, ^29^

When evaluating individuals’ quality of life, it is important to look at their functions in many fields. Strength, energy and ability can help the person to integrate in daily life. Psychological functioning, which is an appropriate field for psychologists, is frequently problematic for physicians and common evaluations drawn from there include anxiety and depression. Psychological functioning can be assessed with a lot of instruments, which may examine relevant aspects and symptoms more likely to be influenced by specific diseases and treatments.^27^ Generic measures of quality of life may fail to address this complexity have the rich and broad range of domains are important for people with mental health problems.^30^

The precautions taken for individuals with mental health problems will affect their lives in an either negative, or positive manner.^30^ Cases identified as negative life are witnessed in many mental diseases. Schizophrenia, chronic depression, manic-depression, dementia and severe personality disorders are long term illnesses and are encompassed under the field of psychiatric research. The principal focus here is on the symptoms which are measured with QoL assessments, impairments, and disabilities. General population measures of QoL are not sensitive to issues faced by this disabled population. QoL is also related to setting goals for psychosocial therapies and rehabilitation. The major interest of psychiatric rehabilitation should be helping individuals with serious mental illnesses to develop the skills needed to objectively reach adequate living conditions.^27^, ^31^

**Protection and Promotion of Mental Health**

Effective psychiatric rehabilitation requires individualized rehabilitative programs. Governments have a huge role on risk and protective factors as far as mental health is concerned in order to establish the actions required to prevent mental disorders and protect and promote mental health at all stages of life.^27^ Governments must develop a phased and budgeted plan for closing down psychiatric institutions that require long-time staying and replacing them with support for discharged patients to live in the community with their families. We must study on community-based mental health services, including outreach services, home care and support, emergency care, community-based rehabilitation and supported housing.^26^

Schoen Makers et al. reported that formal support to caregivers reduces the burden and depression that they experience and emphasized that formal support is an important issue that must be kept in mind. Therefore, it can be assumed that patients receiving home care reduce caregivers’ burdens as well as the rate of depression that brings along many problems, which brings us to the conclusion that policies related to home care services and its feasibility are very important. Like in USA, Canada and some European countries, legal regulations related to home care for psychiatric patients must be established and expanded actively throughout the country.^2^

There are differences between the hospital and home care nursing, one of which is the measure of ability to assess the rate of adverse event soccurring after the services are provided. The emergence of developing adverse effects can be questioned and latergivena more systematic study of hospital care and be subject to the audit service. Care at hospital can be as short asjust a few days, while receiving care at home usually takes a more extended period of time and this situation affects the rate of adverse effects and may extend to many more years.^12^

Blais et al. evaluated the adverse events and ratesduring the home care and the results presented the injuries (17%), wound infections (14%), psychosocial, behavioral, mental problems (11.8%), and other (57.2%) kinds of adverse events occurring during this time^12^. Besides, Seitz et al. have examined 74 studies to determine the psychiatric problems in patients that have taken long-term care, and at the end found that dementia, depression, and anxiety disorders are the most common psychiatric disorders.^32^

Tasdelen and Ates indicate in their first study that patients who took care at home generally had psycho-social problems (72.9%) and the indicators were sadness, anger, helplessness, constant crying, hopelessness, anxiety, introversion, role losses related to work and family life, decreased self-esteem, fear of death, reductions in self-sufficiency, worry caused by depending on someone, continuous presentation of adepressiveimage and social isolation.65% of patients stated that they had experienced sleeping problems and pain, and 53.1% of the patients stated they had problems in terms of receiving drugs and were unable to possess medical supplies, experienced failure in benefiting fromtransportation, had problems with financial issues, were unable to participate in social activities and reaching medical staff.^6^

Consequently, patients with chronic mental diseases as well as their families are influenced by the diseases. To reduce these effects, patient and their families must be supported with home care they can live on. It has been seen in many studies that home care services and psychosocial attempts increase the life quality of patients and their families, besides also reducing costs and symptoms of depression. Home care services and psychosocial attempts must be enhanced, and it is important to work with nursing homes and regulate the policy about such services.

**Limitations of Homecare**

There are some limitations of homecare about qualifications for provision of homecare. If the patient is so psychotic, if he/she has a risk to harm him/herself or any other person, if he/she lives alone, if the physical environment is not convenient, it is recommended that these kind of patients must be institutionalized and take hospital care.^11^

The health care team providing home-care services must read and sign the treatment protocol that is made with the patient and family. During the home visit, the team must be sincere, but also must preserve the professional border.^19^, ^33^ In home-care, cognitive therapy, family therapy and behavioral methods that are applied towards developing self-conception, increasing insight, helping to recognize and acknowledge feelings, increasing motivation for cooperation with team are among the psychosocial interventions.^34^, ^35^, ^36^

### Psychopathology of patient on caregivers

Patients having chronic psychiatric disease are suffering from many problems such as burnout and desperation feelings, they are unable to receive any support or take education about the patient and the disease, and, at these periods, they need professional support. Preparation of educational programs towards families and to plan an individual approach method specific for the condition the patient has with their family is very important. It is expected that providing these approaches with applications will solve many problems that caregivers of patients having psychiatric disease encounters^1^, ^3^, ^26^, ^37^ As known in the literature: Depression symptoms of caregivers should be reduced and should feel less burdened in the home care.^2^ Also it is a way of problem solving and supporting the patients and their families.^17^, ^18^, ^36^

### Limitationsof this review

When we look at the researches made about this subject, it is seen that there aren’t enough studies that examines the impact of the home-care provided to the patients having psychiatric disease on life quality. Thus, it is determined that more studies should be conducted on this subject. Less research, however, has focused on how these interventions should be delivered and whether it is more beneficial to provide them in patients’ homes than in institutions.^18^
